# Reconstructed lung doses for the million person study cohort of 26,650 Tennessee Eastman corporation workers employed between 1942 and 1947

**DOI:** 10.1088/1361-6498/acb1be

**Published:** 2023-02-15

**Authors:** Michael Bellamy, Keith Eckerman, Lawrence Dauer

**Affiliations:** 1 Department of Medical Physics, Memorial Sloan Kettering Cancer Center, New York, NY, United States of America; 2 Oak Ridge National Laboratory (Retired), Oak Ridge, TN, United States of America

**Keywords:** dose reconstruction, epidemiology, lungs, uranium, inhalation

## Abstract

Tennessee Eastman Corporation workers were exposed to uranium dust resulting in high-linear energy transfer (LET) irradiation to lung tissue. In this work, radiation lung doses were reconstructed for 26 650 men and women working at the plant between 1942 and 1947. Site air monitoring data of uranium concentrations and payroll records were used to determine the daily inhaled activities and annualized lung doses. Variations in the activity median aerodynamic diameter of the uranium dust, the solubility of particulate matter in the lungs and the sex-specific breathing rate were investigated as part of a sensitivity analysis. Male and female mean lung doses of 18.9 and 32.7 mGy, respectively, from high-LET alpha irradiation, and there was general agreement with evaluations from previously published epidemiological studies. Annual lung dose estimates and sensitivity analysis for the 26 650 workers in the TEC cohort have been archived on the United States Department of Energy Comprehensive Epidemiologic Data Resource.

## 1. Introduction

The current system of radiation protection practice is based on the principles of justification, limitation, and optimization, mainly because exposure to ionizing radiation is associated with the subsequent development of human cancers [1]. However, significant uncertainties exist regarding the biological response associated with low doses of ionizing radiation [2]. This knowledge gap can be addressed through epidemiological studies of workers who have been occupationally exposed to ionizing radiation [3]. For example, the Tennessee Eastman Corporation (TEC) was located in Oak Ridge, Tennessee in the United States, and was the site of uranium enrichment activities from mid-1942 to mid-1947. Tens of thousands of TEC workers were exposed to internal radiation hazards from inhaling uranium oxide, uranium chloride, or uranium fluoride [4]. Previous epidemiological studies [5–8] estimated the radiation doses of this worker cohort. However, these estimates were based on outdated dosimetric frameworks and have been applied to small groups of workers, such as only white males or workers selected for a case-control study.

Very few epidemiological studies exist where the workers were exposed to high-linear energy transfer (LET) radiation [9, 10]. However, renewed interest in studying the effect of chronic high-LET exposure on healthy workers motivated the present effort to expand and update the radiation dose estimates for TEC workers [3]. Characterizing the organ doses associated with these groups may lead to a better understanding of the link between occupational radiation exposure and health effects such as the development of radiogenic cancers and ischemic heart disease. Intake of uranium is associated with high-LET radiation because of the alpha emissions [11] at several steps of the nuclear decay process [12]. After the initial exposure, uranium usually resides in the lung and nearby lymph tissue for many years. While in the body, uranium will chronically expose the tissue to high-LET radiation [13]. Therefore, expanding and updating the dose estimates of the TEC worker population is of particular significance because the workers were exposed to the high-LET lung irradiation [14]. The comprehensive dose reconstructions described herein have been utilized in a recent epidemiologic study of this population [14].

## 2. Materials and methods

Lung doses for 26 650 workers employed at the TEC between 1942 and 1947 for at least 90 d were estimated using the latest International Commission of Radiological Protection (ICRP) biokinetic and dosimetric models. Historical occupational records determined worker sex, position, start and end dates, estimated airborne uranium concentration, and enrichment levels [14]. While airborne concentration estimates were available for most workers, airborne uranium concentration levels were imputed when these data were unavailable. Annualized lung dose estimates were calculated for each worker based on the assumption of chronic daily intake associated with each work assignment. A sensitivity analysis was performed to estimate the effects of modifying dosimetric parameters such as particle size distribution and the solubility of the inhaled uranium at various stages of the processing chain.

**Figure 1.**
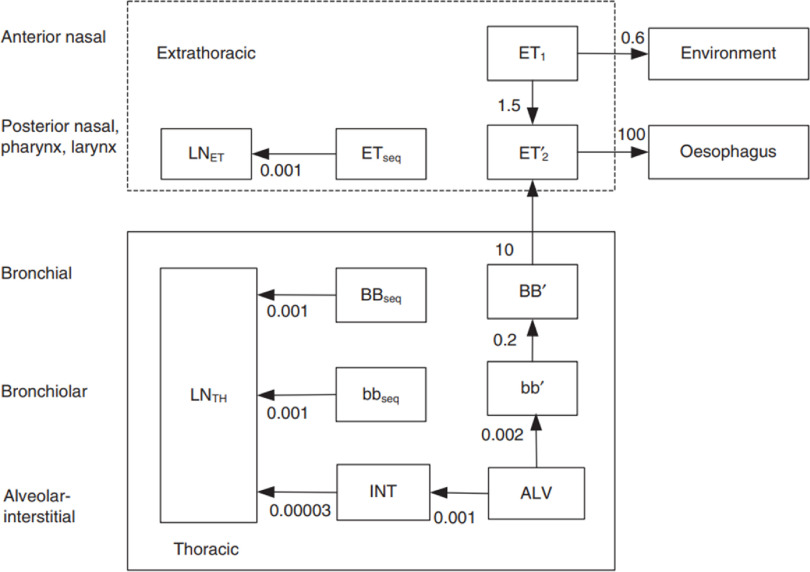
Compartment model representing time-dependent particle transport from each respiratory tract region in the HRTM. The rates shown alongside arrows are reference values in units of d^−1^. It is assumed that 0.2% of material deposited in the posterior nasal passage, pharynx, and larynx (ET_2_), bronchi (BB), and bronchioles (bb) is retained in the airway wall (ETseq, Bbseq, and bbseq, respectively). A fraction (0.67) of the deposit in the ALV region is transfer to the bb’ region with the remainder transferred to the interstitium (INT) where it is slowly transferred to the thoracic lymph nodes.

The updated Human Respiratory Tract Mode (HRTM) of ICRP Publication 103 [1] was applied to calculate the lung dose of former TEC workers. The HRTM addressed two respiratory tissues: the extrathoracic (ET) and the thoracic (TH) airways (figure 1). Subdivision of these tissues focuses mainly on differences in sensitivity to radiation. The TH regions consist of the bronchial (BB) of the trachea and bronchi airways, the bronchiolar (bb) airways, the alveolar interstitial (ALV) gas exchange region, and the thoracic lymph nodes (LN_TH_). The ET region consists of the anterior nasal passage (ET_1_), the posterior nasal passage, pharynx, larynx (ET_2_), and the ET lymph nodes (LN_ET_). The mechanical clearance of deposited particles is shown in figure 1.

The absorption into blood of a radionuclide attached to a deposited aerosol particle depends on the physical and chemical form of the deposited particle. The HRTM assumes absorption occurs at the same rate in all regions (including the lymph nodes), except ET_1_ for which no absorption is assumed. The absorption is treated as a two-stage process: dissociation of the radionuclide from the deposited particles and absorption into blood, i.e. uptake. The rate at which these processes occur can be time dependent.

**Figure 2.**
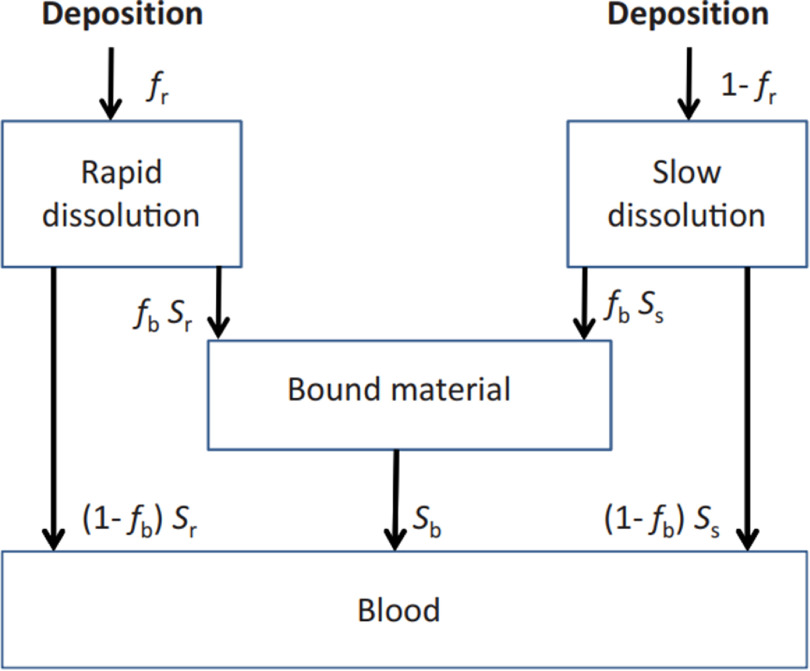
Compartment model representing time-dependent absorption into blood (dissolution and uptake).

HRTM uses a simple compartment model to represent time-dependent dissolution and absorption, as shown in figure 2. It is assumed that a fraction (*f*_r_) of the region deposit dissolves relatively rapidly at a rate *s*_r_. The remaining fraction (1−*f*_r_) dissolves more slowly, at a rate *s*_s_. Uptake into blood of dissolved material is usually assumed to be instantaneous. However, a fraction of the dissolved material might be slowly absorbed into blood because of binding within the respiratory tract tissue. To represent time-dependent uptake, it is assumed that a fraction (*f*_b_) of the dissolved material is retained in the ‘bound’ state, from Ih it enters the blood at a rate *s*_b_, while the remaining fraction (1−*f*_b_) enters instantaneously. Material in the ‘bound’ state is not cleared by mechanical processes but only by uptake into blood. Note that the bound state is not considered, i.e. *f*_b_ = 0 for uranium. The absorption parameters for uranium aerosol with absorption Type M, S, and M/S considered in this work are shown in table 1.

**Table 1.** Absorption parameters for inhaled uranium chemical forms assumed in TEC study^a^.

	Absorption parameter values			Absorption alimentary tract
Absorption Type	*f*_r_	*s*_r_ (d^−1^)	*s*_s_ (d^−1^)	*f*_A_
M	0.2	3	5 × 10^−3^	4 × 10^−3^
S	0.01	3	1 × 10^−4^	2 × 10^−4^
M/S	0.03	1	5 × 10^−4^	6 × 10^−4^

^a^ Values obtained from ICRP Publication 137 [13].

The dose to target cells considered at radiogenic risk within each respiratory tract region is calculated. In the AI region and lymph nodes (LN_TH_ and LN_ET_), the cells are assumed to be distributed throughout these regions as shown in table 2. Within the conducting airways (ET_1_, ET_2_, BB, and bb), the cells are considered to lie in a layer of tissue below the airway surface, and the average dose to this layer is calculated. Within the BB airway, two specific cell types, basal and secretory cells, are considered, in the other regions contain secretory cells. The dose within the AI region (ALV and INT) is averaged over the mass of the lung with its blood content excluding the lymph nodes. The lung dose value is taken as the average of the dose to BB, bb, and AI, with the BB value being the average of the dose to the basal and secretory cells. The dose to the ET airways is the average over ET_1_ and ET_2_ airways.

**Table 2.** Physical parameters for cells considered at radiogenic risk.

			Target mass tissue (kg)	
Region	Target cells	TargetDepth (*µ*m)	Male	Female
BB	Secretory	10–40	8.65 × 10^−4^	7.77 × 10^−4^
	Basal	35–50	5.32 × 10^−4^	3.89 × 10^−4^
bb	Secretory	4–12	1.95 × 10^−3^	1.87 × 10^−3^
AI		^a^	1.100	0.904

^a^ Cells assumed uniformly distributed within the lung and dose averaged over the lung mass, including blood content, less lymph nodes.

Annual lung absorbed doses for each member of the TEC cohort were estimated based on their estimated daily intake of uranium and absorbed dose per unit intake coefficients. Occupational breathing rates for men and women were assumed to be 28.8 and 27.3 m^3^ d^−1^ [15, 16]. The average airborne uranium concentration in Bq per m^3^ was derived and described in previous studies [8, 17]. The extensive information available on the behaviour of uranium after deposition in the human respiratory tract has been summarized in the ICRP Publication 137 on the occupational intakes of radionuclides: Part 3 [13]. Specific parameter values associated with the particulate inhalation of various chemical forms have been assigned into solubility types including Type F, Type M, Type S, Type F/M, Type M/S and a special solubility class for uranium aluminide. For each defined type, absorption parameter values, HRTM transfer coefficients, and *f*_A_ values have been employed to estimate organ absorbed doses [18, 19]. In this work, the three chemical solubilities considered were Type M/S, Type M and Type S. Male lung committed equivalent dose coefficients associated with the inhalation of U-234 in various solubility types and activity median thermodyanic and aerodynamic diameters have been shown in figure 4.

The dosimetric model employed in this work assumed that workers received daily intakes of uranium as described in Carpenter *et al* [20]. The uranium intake for a particular day of employment was derived as the product of the airborne uranium concentration, the daily breathing rate, and the number of hours worked. The set of daily intakes was then multiplied by the appropriate ICRP modelled lung absorbed dose rate coefficient (per unit intake) to determine the lung absorbed doses as a function of time for each assumed daily acute intake. This set of lung absorbed dose rates for all of the individual daily intakes for each worker were then added to determine the worker’s total absorbed lung dose associated with TEC uranium inhalation. Finally, the lung dose rate values were integrated to determine worker doses evaluated for each discrete calendar year.

Historical occupational records form the basis of this study’s dosimetric assessment. Personnel information such as date of hire, term of employment and department were obtained from payroll and human resources information for each employee position. Industrial hygienists at that time employed on-site measured uranium air concentrations by analysing vacuum filters and greased Petri dishes. Alpha particle activity was estimated using ionization chambers, an electrometer and, by a fluorescent method. The distribution of uranium particle sizes was measured at the site along with estimates of filter efficiency. This historical measurement information was available and used in the present dose reconstruction effort. Additional details on the collection of these occupational records have been previously published [14, 17].

Temporal- and location-specific airborne uranium concentration, an essential parameter in estimating internal dose, was available for many but not all workers. To calculate the lung dose for workers with no specific information about their average inhaled uranium concentration, an imputation process was employed, matching these workers to similar workers at co-locations with known concentration estimates. For most cases, there were other workers in the same department with the same job ID number and with the same job title. An algorithm was developed to define sets of workers with known average airborne concentrations that would most closely match a particular candidate. Workers who matched department, job ID, and job title took the highest priority as complete match candidates. The most similar set of workers was chosen to assign average concentration for partial matches. A priority system of weights was used to select worker groups that most closely aligned with the candidate worker. Department code was given the highest weight of four followed by job ID with a weight of two and job name had the lowest weight of one. Job name was assigned the lowest priority because multiple job title text strings of the source dataset occasionally referred to the same job ID (e.g. ‘Janitor’ ‘janitr’ ‘JAN ITOR’) and these variations in job-title were manually updated as part of project quality assurance.

A set of sensitivity analyses was conducted to determine the variation in doses resulting from different dose reconstruction assumptions. The sensitivity analyses included particle size distributions ranging from 1 *µ*m to 10 *µ*m around a central estimate of 5 *µ*m. In addition, several assumptions of solubility were investigated including Type S, M and M/S as defined by recent ICRP recommendations. Finally, variations in breathing rates and workday duration were investigated as described in the epidemiologic study [14].

## 3. Results

**Figure 3.**
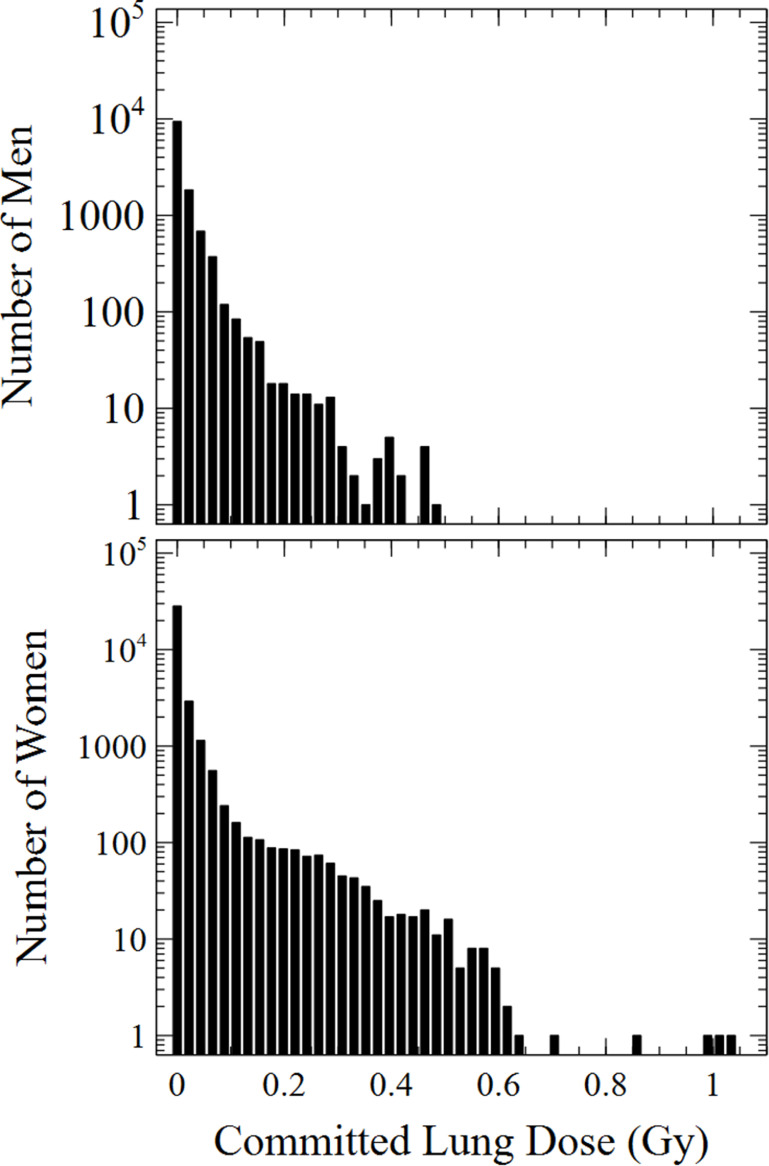
Committed lung doses of 13 951 female and 12 699 male workers employed at the TEC associated with the isotopic separation and enrichment of uranium during the Manhattan project.

A histogram of the committed lung doses of 13 951 female and 12 699 male workers employed at the TEC is shown in figure 3. The doses in this figure were reported under the central, main analytic assumptions of an activity median aerodynamic diameter (AMAD) of 5 *µ*m, a Type M/S solubility of the particulate uranium in the lungs, and a 6 h workday [14]. A right-skewed distribution of committed lung doses was observed, where most workers received rather low lung doses associated with occupational inhalation, while a few workers received lung doses as high as 1048 mGy.

**Figure 4.**
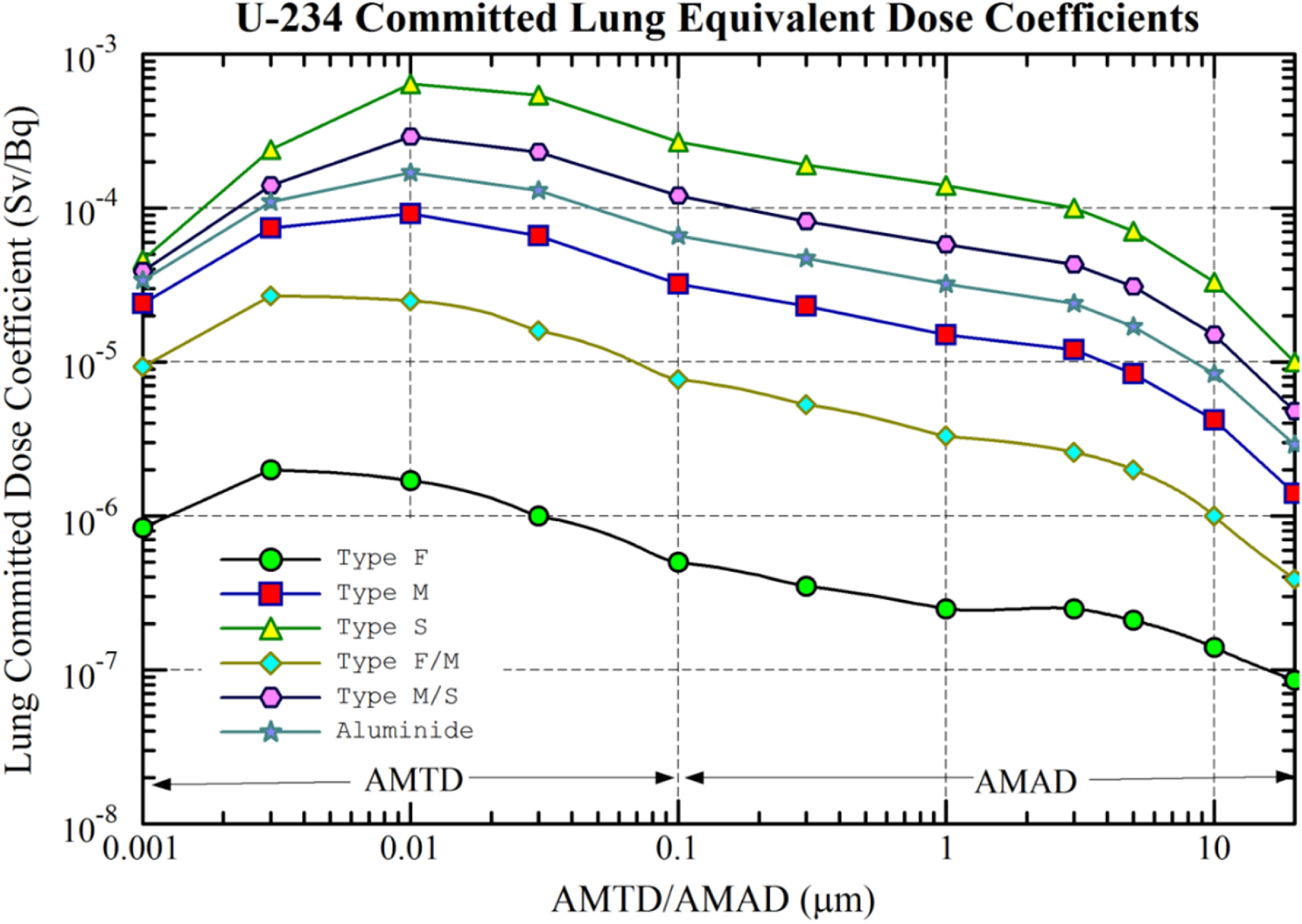
Male lung committed equivalent dose coefficients associated with the inhalation of U-234 in various solubility types and activity median thermodyanic and aerodynamic diameters (AMTD/AMAD) based on ICRP publication 137 occupational intake of radionuclides: part 3.

Table 3 shows the lung doses estimated for workers of the TEC. This table provides descriptive statistics for the doses calculated under various assumptions of particle size distribution (microns), lung solubility of inhaled uranium particles, breathing rates for both worker sexes (m^3^ d^−1^) and the workday length (hours). The number of workers included in this analysis was 26 650 consisting of 13 951 women and 12 699 men.

**Table 3.** Cumulative Lung doses (mGy) for female and male TEC workers for various assumptions of particle size distribution (microns), lung solubility of inhaled uranium particles, breathing rates (m^3^ d^−1^), and the workday (hours).

				TEC female lung doses (Gy)				TEC male lung doses (Gy)			
				(*N* = 13 951)				(*N* = 12 699)			
AMAD (microns)	ICRP 137 solubility type	Male/Female breathing rates (m^3^ d**^−1^**)	Workday (hours)	Mean	Median	Std	Max	Mean	Median	Std	Max
5	M/S	28.8/27.3	6	32.7	7.6	73	1048	18.9	6.3	35	501
10	M/S	28.8/27.3	6	15.5	3.6	34	491	8.9	2.9	16	235
1	M/S	28.8/27.3	6	62	14.3	139	1999	35.8	11.9	66	953
5	M/S	15.26/14.45	6	61.2	14.1	137	1967	35.5	11.8	65	940
5	M/S	54.00/51.11	6	17.5	4.09	39	557	10	3.3	18	265
5	M/S	28.8/27.3	8	43.6	10.1	97	1399	25.2	8.4	46	668
5	M/S	28.8/27.3	4	21.9	5.1	49	700	12.6	4.2	23	334
5	S	28.8/27.3	6	88.4	20.1	201	2903	47.8	15.9	89	1296
5	M	28.8/27.3	6	8.4	2	19	261	5	1.6	9	130

Male and female organ doses followed a highly right-skewed distribution, with small groups of workers receiving much larger doses than the median. This distribution can be seen in table 3, where the maximum doses received were much larger than the mean, median and standard deviation of lung doses. Male and female mean lung doses of 18.9 and 32.7 mGy, respectively, were associated with assumptions of a 5 *µ*m AMAD, M/S solubility class, 28.8/27.3 m^3^ d^−1^ breathing rate and a 6 h workday. Median male and female lung doses, under the same assumptions, were 6.3 and 7.6 mGy, respectively. Unless otherwise indicated, the lung doses reported in this work assume an integration period from the time of exposure to the year 2021.

**Figure 5.**
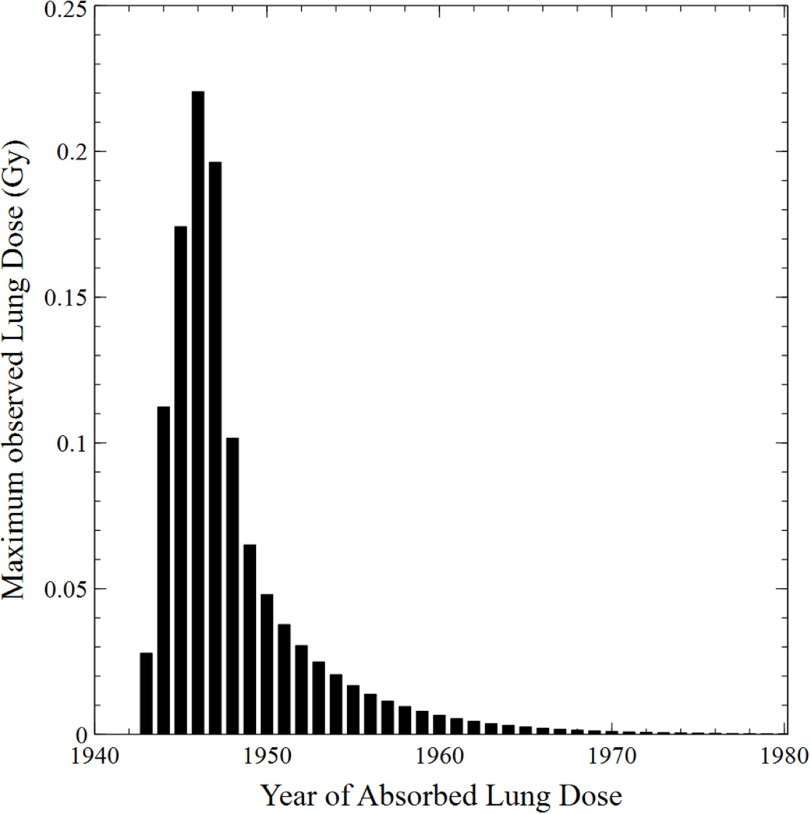
Illustrates the maximum observed annualized lung doses for male and female workers in the TEC cohort between 1940 and 1980. The highest dose of 0.22 Gy y^−1^ was observed in 1946, while doses lower than 0.0001 Gy yr^−1^ were observed after 1981.

The maximum lung doses for each year are shown as in figure 5. The largest observed lung dose for any single year was observed in 1946 and was 0.22 Gy y^−1^. Overall, the lung distribution exhibited a left-skewed distribution because the period of radioactive dust exposure occurred over 4 years and because the inhaled material was biologically removed from the lungs at a reasonably fast rate. Although the plot ranged from 1940 to 1980, there a small but finite dose rate lower than 0.0001 Gy y^−1^ beyond 1981.

The significant dosimetric model parameters were varied as part of sensitivity analyses [14]. Increasing the AMAD from 5 to 10 *µ*m was associated with a two-fold decrease in lung dose. Decreasing the AMAD from 5 to 1 *µ*m was associated with a two-fold increase in lung dose. Changing the assumed solubility of the inhaled uranium particles from M/S to S was associated with a four-fold increase in lung doses. Conversely, increasing the assumed solubility of the inhaled uranium particles from M/S to M was associated with a three-fold decrease in lung doses. These results are consistent with the trends in the committed lung doses of ICRP Publication 137, delineated by particle size distributions and solubility classes, as summarized in figure 4.

The entire set of lung dose estimates has been anonymized and uploaded to the United States Department of Energy (USDOE) Comprehensive Epidemiologic Data Resource (CEDR). This publicly available electronic data repository is comprised of health studies of DOE contract workers and environmental studies of areas surrounding DOE facilities. The TEC dosimetry database and cohort mortality outcomes are hosted on the TEC section of CEDR website [21].

## 4. Discussion

Although the lung doses estimated in this study are comparable with several previously published works, there are a few key differences. The primary difference is that the doses considered in this work have been calculated as annualized (i.e. lung absorbed dose in a given year) instead of committed lung doses. Additionally, this study estimates lung doses for groups beyond the narrower cohort definitions of previous studies. Women were found to receive higher mean and median lung doses than men because they were observed to stay in high uranium concentration jobs for longer periods of time than their male counterparts.

Lung cancer among workers at the TEC was examined in 1983 by the Department of Cancer Control and Epidemiology [22] as a case-control study involving white males who received radiation to the lungs from uranium dust inhalation. This case-control study reported that the cumulative radiation dose to the lungs ranged from 0 to 0.75 Gy. Workers with a cumulative lung dose of 0.2 Gy and higher were associated with statistically significant increases in lung cancer risk. Workers were broken up into groups based on cumulative lung dose levels. The workers were stratified based on levels of exposure into the following categories: unexposed, low, medium, and high dose groups, and were further stratified by age into subgroups younger and older than 45 years. The range of committed lung doses of the white male TEC workers falls well within the range of doses estimated in the sensitivity analysis of this study.

Lung doses for TEC men were estimated based on a 30 year commitment period as part of a more extensive epidemiology study [8]. These lung doses were calculated based on exposure to uranium dust based on time spent at the plant, the solubility of inhaled material, isotopic enrichment, and airborne concentrations of uranium. Lung doses were calculated considering the fraction of inhaled material deposited in the pulmonary region, the total inhaled activity as a function of time, and the dose rate per unit time and unit activity of the specific uranium isotopes in the lung. A single clearance rate was chosen based on the effective half-life of the uranium compound in the lungs. The assumed radionuclide lung deposition fraction was 15%. Biological half-lives for uranium oxides, uranium fluoride, and uranium chloride were 500, 250, and 100 d, respectively. These values have been updated in the biokinetic model used in the current study [13]. A single breathing rate of 10 m^3^ per day, along with an assumption of 250 workdays per year, was assumed to derive the total annual intakes. This study estimated that the lungs of these workers might have been exposed to doses as high as several Sieverts when considering radiation weighting factor of alpha emitters.

The relationship between lung cancer mortality and uranium dust exposure among workers in four uranium facilities, including the TEC, was reported in another previous study [7]. In the second case-control study, air monitoring data from general area detectors identified by date and location were used to estimate average uranium dust concentrations. These exposure concentrations, along with the number of days worked were used to estimate annual integrated activities in the lung, which was then used to calculate the radiation dose to the lung. A matched case-control study design was employed with 428 pairs of workers employed at the TEC. The authors reported an odds ratio of 2.0 for workers exposed to 25 cGy or higher. However, they noted a very large confidence interval around that estimate. A direct comparison between the doses published by Dupree *et al* [7] and the current work was not possible as their worker-specific records were unavailable.

The sensitivity analyses conducted in this work shows that dosimetric assumptions are significant when evaluating lung doses. The solubility of inhaled radioactive material has a greater impact on radiation dose to the lungs than particle size distribution, as it affects the rate at which the material is cleared from the lungs. Highly soluble material is more likely to be absorbed into the bloodstream and leave the lungs rapidly, thus reducing the amount of time that it is exposed to sensitive lung tissue and resulting in a lower radiation dose. Particle size also has an effect on how deeply the particles are deposited in the lungs; smaller particles can penetrate further, increasing their exposure to sensitive lung tissue and leading to a higher radiation dose. However, even if a particle is small enough to penetrate deeply into the lungs, if it is soluble, it will remain in the lungs for a shorter period of time, decreasing its exposure to sensitive lung tissue and resulting in a lower radiation dose. Therefore, solubility is more influential than particle size when assessing radiation dose to the lungs, as it affects how quickly the material is cleared from the lungs. Highly soluble material leaves the lungs relatively quickly, reducing its exposure to sensitive lung tissue and generally resulting in a lower radiation dose than small particle sizes. The relative importance of solubility to particle size distribution has been shown in figure 2. However, particle size can be of significant concern when addressing the dose over short periods, e.g. five years. Thus, both aerosol size and chemical form of uranium in the lungs are essential considerations for this worker cohort. Several data and dosimetry limitations exist in the scientific basis of this work. Air monitoring data were used to estimate worker intakes instead of urinalysis data that was not available for this cohort. Workers’ primary physical location was assumed to correspond directly to payroll job titles and buildings. Occupational medical radiography records were not available for this study. Personnel-level external dosimetry was unavailable because film badges were not used at the site at that time. There was insufficient information about respirator usage to include a protection factor in the lung dose calculations [23, 24].

The most important limitation of this work is the reliance on air monitoring data to estimate worker intakes. Urinalysis is the most accurate dosimetry data source; these records should be employed whenever possible. However, in the TEC cohort, urinalysis records were unavailable, so individual dose reconstructions were based on air monitoring results. While the precision of the dose reconstruction would undoubtedly be lower than if it were based on individual urinalysis, a reconstruction based upon air monitoring data was the only viable method for this cohort. In addition, using air monitoring data when no bioassay data are available has been previously recommended [25] based on reported positive correlations between air monitoring results and urinalysis results for uranium workers [26].

Several relevant variables were checked in the Polednak *et al* [8] study from original TEC payroll records. All chemical workers were verified as having worked in chemical departments, and only a few significant errors were found. However, the payroll records were not intended for dose reconstruction, so there may not always be a direct correspondence between the worker’s payroll department and job title and the assigned air monitoring results.

Personnel badges were not available to estimate external dose to the TEC worker cohort, and external dose was not considered in this study. However, since uranium and progeny are primarily alpha emitters, external lung doses were expected to be much smaller than the corresponding internal doses. Moreover, while some uranium ores being processed in the 1940’s were active gamma sources, these ores were not handled at TEC. However, they were processed earlier in the manufacturing chain at Mallinckrodt Chemical Works [27]. Therefore, the exclusion of external doses is not likely to affect the lung dose estimates for the TEC worker cohort.

## 5. Conclusions

A large cohort of young women and men were employed by the TEC between 1942 and 1947 and were involved in enrichment activities for World War II. Many were chronically exposed to airborne uranium associated with the military plant’s electromagnetic enrichment mission during their employment. This work reconstructed lung doses for 13 951 women and 12 699 men based on air monitoring data and occupational site records. The latest ICRP dosimetric and lung models were employed to determine annualized lung doses associated with inhaling various chemical forms of uranium at the site. Median male and female lung absorbed doses were 6.3 and 7.6 mGy, respectively, while mean male and female lung doses were 18.9 and 32.7 mGy respectively. Sensitivity analyses were conducted to explore uncertainty bounds by estimating worker lung doses associated with variations in AMAD, solubility type, breathing rates, and the number of hours worked per day. There was general agreement in the lung dose estimates based on main analytic assumptions of an AMAD of 5 *µ*m, a Type M/S solubility of the particulate uranium in the lungs, and a 6 h workday with lung dose evaluations from previously published epidemiological studies. The dose estimates of this cohort can be used to support radiation epidemiological studies on high-LET irradiation associated with chronic occupational intake [10, 14].

## Data Availability

The data that support the findings of this study are openly available at the following URL/DOI: https://oriseapps.orau.gov/CEDR/search_results.aspx?DataSet=TEB22A01.
